# The Efficacy of Green Tea Chewing Gum on Gingival Inflammation

**Published:** 2016-06

**Authors:** Parichehr Behfarnia, Ahmad Aslani, Foroogh Jamshidian, Soheil Noohi

**Affiliations:** 1 Dental Implant Research Center and Dept. of Periodontics, School of Dentistry, Isfahan University of Medical Sciences, Isfahan, Iran.; 2 Dept. of Pharmaceutics, School of Pharmacy and Novel Drug Delivery Systems Research Center, Isfahan University of Medical Sciences, Isfahan, Iran.; 3 Professional Doctorate, School of Dentistry, Isfahan University of Medical Sciences, Isfahan, Iran.; 4 Postgraduate Student, Dept. of Periodontics, School of Dentistry, Isfahan University of Medical Sciences, Isfahan, Iran.

**Keywords:** Green Tea, Gingivitis, Plaque Index

## Abstract

**Statement of the Problem:**

According to previous studies, the components of green tea extracts can inhibit the growth of a wide range of gram-pos-itive and -negative bacterial species and might be useful in controlling oral infections.

**Purpose:**

The aim of this study was to determine the effect of green tea chewing gum on the rate of plaque and gingival inflammation in subjects with gingivitis.

**Materials and Method:**

In this double-blind randomize controlled clinical trial, 45 patients with generalized marginal gingivitis were selected and divided into two groups of green tea (23) and placebo (22) chewing gum. The patients chewed two gums for 15 minutes daily for three weeks. Sulcus bleeding index (SBI) and approximal plaque index (API) were studied at the baseline, 7 and 21 days later. Saliva sampling was conducted before and after 21 days for evaluation of IL-1β. The results were analyzed and compared by using repeated measures ANOVA, paired t test, and independent two-sample t test (α=0.05).

**Result:**

The results showed that chewing gum significantly affected the SBI and API (*p*< 0.001). Paired t test showed that the two groups were significantly different regarding the mean changes of SBI and API at different periods of 1-7, 1-21, and 7-21 (*p*< 0.001). Concerning IL-1β, the repeated measures ANOVA revealed that the effect of chewing gum was significant (*p*<0.001). Moreover, paired t-test represented no significant difference between the mean changes of IL-1β within 1-21 day (*p*= 0.086).

**Conclusion:**

The green tea chewing gum improved the SBI and API and effectively reduced the level of IL-1β.

## Introduction

During the recent century, a main advancement has been attained in utilizing the herbs such as tea in medicine. Generally, three types of tea including black tea, green tea and Oolong tea (a Chinese type of tea) are derived from a shrub named *Camellia sinensis*. Green tea has advantage to other types of tea because it is less influenced by the fermentation process and its compositions remain stable. Green tea is known as an anticancer and antioxidant compound.[1-3]

Many properties of the green tea are related to a component called catechin whose anti-oxidation activity is very pronounced and acts much stronger than other antioxidants such as vitamins C and E.[4]

In a study,[5] it was observed that the polyphenols of green tea like Epigallocatechin gallat (EGCG) acted against TNFα and IL-1β inflammatory mediators and reduced the production of inflammatory cytokines such as IL8, which had an important role in absorbing neutrophils to the inflammatory sites. A number of researches on the green tea demonstrated that it has an anti-bacterial property against gram-positive and gram-negative bacteria such as *Prevotella intermedia* and *Prevotella nigrescens*. It also prevents the binding of *porphyromonas gingivalis(P. gingivalis)* bacteria to oral mucosal cells.[4-6]

In addition, the catechins of green tea have the esteric structure of galloy radial, epigallocatechin gallat, and gallocatechin gallate which are the polyphenols that prevent the production of toxic metabolites of P. gingivalis.[7] These compounds prevent the formation of biofilms that contain *P. gingivalis* and *Fusobacterium nucleatum (F. nucleatum)*, as well as the binding of periodontopathogens; they also reduce periodontal destruction.[8-9]

One of the most important properties of green tea polyphenols is their restrictive effect on oxidative reactions of reactive oxygen species (ROS). ROS include free radical derivatives of oxygen. Green tea reduces the secretion of lysosome by separating ROS from the transition metal ions and prevents Fenton reactions and/or catalyzing oxidation reactions of the other molecules.[10-12]

Since there was no completely similar study concerning the impact of green tea as chewing gum on oral diseases and gingival inflammation, conducting such a study seemed to be necessary. The objective of this study was to demonstrate the effect of green tea chewing gum on the level of plaque, gingival bleeding, and inflammation after 21 days consumption in patients with gingivitis. 

## Materials and Method

This double-blind randomized controlled clinical trial was conducted on 45 subjects suffering from generalized marginal gingivitis who referred to Isfahan School of Dentistry in 2013. The selected patients had an age range of 30-40 years. The research objectives were explained to the patients and informed consent was obtained. During the experimental period, none of them received medical intervention which might influence the outcome of the study. The design of the study was based on the study conducted by Krahwinkel and Willershausen.[13]

As the inclusion criteria, the patients were supposed to be cooperative enough and had not received any periodontal treatment in the recent past six months. Those who suffered from systemic and metabolic diseases, used medications that could influence the intervention (chewing gum), had less than 20 teeth, pregnant patients, and the smokers were excluded.

After initial examinations, the sulcus bleeding index (SBI) and approximal plaque index (API) were measured, salvia samples were collected, and the patients were divided into experimental (G, n=23) and control (P, n=22) groups. The patients in G group were given two green tea chewing gums each day and those in P group received two placebo chewing gums each day for 21 days. All clinical indices were measured at the beginning, day 7, and day 21. The patients were instructed to chew a gum for 15 minutes twice a day at least one hour before meal, once in the morning and once in the evening or at night. Out of the total of 2100 chewing gums, half were green tea chewing gums and the other half were placebo, with respect to consumption of which the patients were allocated to either group.

Green tea chewing gum and placebo chewing gum were prepared by the Faculty of Pharmacology, Isfahan University of Medical Sciences. Both types of chewing gum had the four bases of Elvasti, Prosty, 487, and stick. The sweeteners used in chewing gums were maltitol and xylitol. The green tea chewing gums used in this study were selected based on the published study of Aslani *et al.*[14] The green tea extract used in this survey contained 207.32 mg/g caffeine, 130 mg/g catechin, and 200.82 mg/g flavonoid. It should be noted that the rate of these elements was different in various areas. Totally, the rate of green tea used into each green tea chewing gum was 120 mg.[14] The standard rate of green tea is 240-320 mg polyphenol or 100-750 mg green tea per day.[4, 15] Before beginning the study, the patients were instructed to brush their teeth daily by using fluoride toothpaste. Seven days later, API and SBI were measured in all patients.

To measure the API by using a detector pill, the buccal surface of upper left and lower right quadrant teeth and the lingual surface of upper right and lower left quadrant teeth were investigated for the presence of plaque. Then, the percentage of API was calculated based on proportion of the number of areas with plaque to the total areas of measured values.[16]

In order to measure the SBI, the buccal and lingual marginal gingival and distal and mesial surfaces of papillary gingiva of the total teeth were studied (four surfaces of each tooth). The SBI value was described 30 seconds after gently inserting a periodontal probe into the sulcus of gingiva. It was scored depending on the rate of bleeding. Finally, the SBI was obtained through dividing the number of points with bleeding by the total studied points.[17]

In order to evaluate the IL-1β at the first and third session (21 days later), salvia samples were collected before breaking the fast at morning. Patients were asked to keep their mouth relaxed for 5 minutes and then spout into a glass. This saliva was utilized in order to measure IL-1β inflammatory factor (unstimulated method). Then, the level of IL-1β was measured by using ELISA (IL-1β kits; Orgeniom Co., Indonesia).

The patients were asked to use two chewing gums daily (each gum for 15 minutes, totally 30 minutes). The API and SBI were measured after 7 and 21 days. The IL-1β was measured again after 21 days. Data were analyzed using brief statistical indices including the mean, standard deviation (SD), repeated measures ANOVA, paired t test, and independent two-sample t test (α=0.05). 

## Results

**Figure 1:**
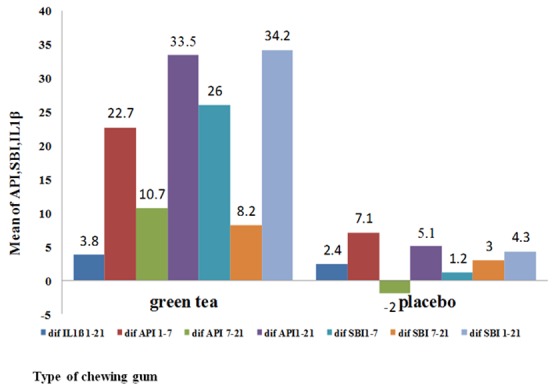
Comparing the mean differences over the given timespans

In this study, 45 participants completed the study process. The indices were not significantly different between the groups at the baseline (*p*< 0.001). Table 1 shows the mean±SD of API, SBI, and IL-1β of the groups in days 1, 7, and 21. Repeated measures ANOVA showed that the API was significantly affected by the chewing gum type (*p*≤ 0.001) (Table 1). Table 2 and Figure 1 display the changes of mean± SD of different indices in the two groups over the given timespans.

**Table 1:** Mean±SD of SBI, API, and IL-1β indices

**Timespan** **Mean±SD of Indices (%)**		**Day 1**	**Day 7**	**Day 21**	**P.Value**
API	Green Tea	46.16±13.10 (n=23)	23.37±8.12 (n=23)	12.65±8.75 (n=23)	<0.001
	Placebo	45.26±11.53 (n=22)	38.09±8.12 (n=23)	40.12±0.45 (n=22)	0.004
SBI	Green Tea	46.39±22.91 (n=23)	20.37±12.44 (n=23)	12,12±10.84 (n=23)	<0.001
	Placebo	45.35±14.36 (n=22)	44.10±10.53 (n=22)	41.04±9.88 (n22)	
IL-1β	Green Tea	7.83±2.64 (n=23)	-	3.99±2.52 (n=23)	0.038
	Placebo	8.81±3.89 (n=21)	-	6.40±2.53 (n=21)	

**Table 2:** Comparing the reduction of indices (differences) between the study groups in the given timespans

**Indices**	**Group**	**dif 1-7**	**dif 1-21**	**dif 7-21**
API	Green tea placebo	*p*< 0.001	*p*< 0.001	*p*< 0.001
SBI	Green tea Placebo	*p*< 0.001	*p*< 0.001	*p*< 0.001
IL-1β	Green tea Placebo	----	*p*< 0.086	-----

**API changes**

The mean±SD of changes of API over the first 7 days (days 1-7) was 22.79±10.90 in G group and 7.17±7.86 in P group; over the whole period of the study (days 1-21), it was 33.5±7.38 in G group and 5.14±11.08 in P group; over the 7-21 days timespan, it was 10.71±7.38 in G group and -2.03±3.31 in P group. T test revealed a significant difference in mean±SD of changes in API of both groups in these given timespans (*p*< 0.001) (Table 2, Figure 1).

**SBI changes**

Repeated measures ANOVA showed that the type of chewing gum significantly affected the SBI (*p*< 0.001) (Table 1). The mean±SD of SBI changes in 1-7 days timespan was 26.02±10.47 in G group and 1.25±3.83 in P group. In 1-21 days’ time span, the mean±SD changes of SBI was 34.26±19.77 in G group and 4.31±4.48 in P group, and finally, this value at the given time period of 7-21days  was 8.24±6.50 in G group and 3.06±0.65 in P group. T test revealed that the groups were significantly different regarding the mean±SD of changes in SBI in the given timespans (*p*< 0.001). (Table 2, Figure 1).

Repeated measures ANOVA performed on IL-1β showed that it was significantly affected by the chewing gum type (*p*=0.038). (Table 1) The mean±SD of IL-1β changes over the whole 1-21 days was 3.83±2.34 in G group and 2.41±3.02 in P group. T test was used to compare the two groups in terms of the mean changes of IL-1β over 1-21 days. They were found to have no significant difference at the significance level of 0.05 (*p*= 0.086). (Table 2, Figure 1)

## Discussion

Pathogenesis and treatment of periodontal diseases has undergone particular changes during the last decades. Undoubtedly, non-surgical therapies have an important role in reducing bacterial plaque and repairing the tissue. Currently, in non-surgical and non-mechanical (scaling and root planing) therapies, plants extract, coenzymes, and specific vitamins are used for strengthening and reinforcing the oral tissues and the host's resistance.[18] The extract of plants such as green tea has been always used as mouthwash, and a composition of toothpaste or candies at the periodontal maintenance phase. To the best of the authors’ knowledge, the current research was the first study designed to investigate the reaction of inflammatory gingiva after using green tea chewing gum. The two most important findings were that using green tea chewing gum significantly decreased the API and SBI indices that are indicators of gingivitis, as well as the level of IL-1β as a pre-inflammatory cytokine in salvia. During 21 days, using green tea chewing gum caused more alleviation of gingivitis in the experimental group. Using placebo chewing gum for 7 days slightly reduced the gingivitis in the control group, when continued however; no more reduction was observed. The findings supported the positive effect of green tea in decreasing gingivitis after one and three weeks. The obtained results, relating to plaque index improvement and decreased bleeding, were consistent with the results of previous studies conducted by Taylor *et al.*,[6] Krahwinkel and Willershausen,[13] Paveto *et al.*[19] Although, Kuvda *et al.* did not report a change in gingival index after using green tea mouthwash for 3 weeks.[20] This discrepancy might have been due to the different models for using green tea. The continuous flow of gingival crevicular fluid levels and the washing property of salvia may reduce the effectiveness of mouthwash; however, chewing the gum for about 15 minutes would preserve an effective substance in the mouth which would probably not be eliminated by the salivary flow within a short time.

IL-1β is recognized as a pathologic and pre-inflammatory factor since its level increases in salvia and gingival crevicular fluid during the progression of gingivitis. The present study showed that using green tea lowered the level of IL-1β. This finding was consistent with the results found by Maruyama *et al.*[21] and Nakamura *et al.*[22] Oxidative stresses play an important role in the initiation and progressing of periodontal inflammation. Green tea catechins reduce oxidative stresses by decreasing the production of pre-inflammatory cytokines. The two aforementioned studies reported that the green tea catechins decreased the production of IL-1β in gingival tissue as a response to lipopolysaccharide. It suggests that the green tea catechin relieves the oxidative stress as a modifier of innate immunity system.[21-22]

Kushiyama *et al.* noticed a reversed relation between consuming green tea and periodontal disease.[23] Moghbel *et al.* concluded that green tea mouthwashes reduced the rate of anaerobes bacteria more than chemical mouthwashes; it also prevented halitosis and plaque formation.[24] The positive trend of plaque reduction in this study may be related to the antibacterial property of green tea polyphenols. Kaneko *et al.* [25] found that a 4-week mouthwash regimen with diluted catechin solution reduced halitosis and periodontal disease. Green tea catechins especially EGCG (which is active at 250-500 mg/ml concentrations) restrained the growth and adhesion of *P. gingivalis* to the epithelial cells of buccal surfaces. Labrecque *et al.* stated that polyphenols at ≥50 µg/ml concentration can prevent the attachment of *P. gingivalis* to oral epithelial cells and the surfaces covered by extracellular proteins.[8] According to the study performed by Bonifait and Grenier, green tea compositions prevented the formation of *P. gingivalis* and *F. nucleatum* biofilm, and restrained the binding of periodontopathogens to the surfaces.[9]

There is no completely similar research about the effect of green tea chewing gum on the oral diseases and gingival inflammation. However, some researchers studied the influence of chewing gums made of other plants on gingival inflammation. The present study proved that using both chewing gums decreased the plaque index, bleeding index, and IL-1β. The decrease in indices in placebo group is probably due to the chewing practice, saliva  secretion, increased flow of gingival crevicular fluid, as well as its washing and anti-inflammatory properties. But, the decreased rate of these indices in experimental group was significantly higher compared with the control group. Comparing the two groups regarding the mean reduction of indices over the given time spans revealed significant differences in the indices between experimental and control groups. It suggested that in addition to chewing practice, the anti-inflammatory property of green tea could decrease plaque and bleeding indices significantly in experimental group. With respect to the previous studies and the current one, it may be claimed that green tea reduces not only the bleeding of gingiva and formation of plaque that causes gingivitis, but also the gingival inflammation. Furthermore, using green tea in form of chewing gum has advantages over other medication forms because more people are willing to use this medication form, it is convenient to be carried out, it can be used for both patients and non-patients, and it is non-toxic to children and pregnant women. On the other hand, compared with the green tea chewing gum, applying green tea in form of mouthwash or toothpaste provides a shorter contact time between this substance and the gingiva. Thus, it can be concluded that the longer persistence of this medication form in vicinity of the gingiva can increase its anti-inflammatory effect. The most prominent superiority of chewing gum to other medication forms is its effectiveness in both mucosal and systemic forms. Moreover, the systemic absorption of the green tea contents of this chewing gum has positive effects. Accordingly, green tea chewing gum can be easily used daily as a preventive method to reduce the probability of gingivitis or as a mechanical method after treatment of gingivitis to reduce the likelihood of disease recurrence. A further study in larger scale is warranted for better evaluation green tea chewing gum effects.

## Conclusion

The results of this study indicate that applying green tea as a chewing gum improves plaque and bleeding indices and also reduces IL-1β which is a pro-inflammatory cytokine. Therefore, the green tea chewing gum may be a safe adjunct for treatment of gingival inflammation. 
